# Three-Dimensional Characterization of Mechanical Interactions between Endothelial Cells and Extracellular Matrix during Angiogenic Sprouting

**DOI:** 10.1038/srep21362

**Published:** 2016-02-23

**Authors:** Yue Du, Sahan C. B. Herath, Qing-guo Wang, Dong-an Wang, H. Harry Asada, Peter C. Y. Chen

**Affiliations:** 1 Department of Mechanical Engineering, National University of Singapore, Singapore; 2 Department of Electrical and Electronic Engineering Science, University of Johannesburg, South Africa; 3 Division of Bioengineering, Nanyang Technological University, Singapore; 4 BioSystems and Micromechanics Interdisciplinary Research Group, Singapore-MIT Alliance for Research and Technology Program, Singapore; 5 Department of Mechanical Engineering, Massachusetts Institute of Technology, USA

## Abstract

We studied the three-dimensional cell-extracellular matrix interactions of endothelial cells that form multicellular structures called sprouts. We analyzed the data collected *in-situ* from angiogenic sprouting experiments and identified the differentiated interaction behavior exhibited by the tip and stalk cells. Moreover, our analysis of the tip cell lamellipodia revealed the diversity in their interaction behavior under certain conditions (e.g., when the heading of a sprout is switched approximately between the long-axis direction of two different lamellipodia). This study marks the first time that new characteristics of such interactions have been identified with shape changes in the sprouts and the associated rearrangements of collagen fibers. Clear illustrations of such changes are depicted in three-dimensional views.

Angiogenesis is the process of forming new blood capillaries from preexisting ones. Activated by the angiogenic growth factors, endothelial cells (ECs) resting on capillary walls begin to release enzymes that degrade the capillary basement membranes. The ECs then invade the extracellular matrix (ECM) and form sprouts in which the leading cells are called tip cells and the others are called stalk cells. Maturing sprouts fuse with neighboring sprouts to form capillary loops. Physiological angiogenesis is crucial to wound healing and the formation of granulation tissue, whereas pathological angiogenesis has been recognized in various illnesses such as cancer, stroke, arthritis and psoriasis^1^,^2^.

The mechanical interactions between a cell and the ECM generally refer to the cell-mediated assembly of ECM proteins and the subsequent cellular responses to the ECM’s resistance to deformation, such as the reshaping of lamellipodia and the changing of cellular attachment to certain ECM regions. Extensive studies have explored such interactions as they occur on a cell-substrate interface. The findings in^3^ revealed that the cell-substrate interaction proximal to the cell boundary is more pronounced, appearing as a pull action with a direction that is highly consistent with the inward normal of the boundary contour. In addition, pushes acting on the substrate, whether almost balanced or imbalanced in the anterior and posterior regions of the cell, have been suggested in^4^. Although these findings establish exact forms of cellular interaction with a substrate, they may not generalize well when elucidating situations in which cells are encapsulated by the ECM. This is evidenced by the fact that in terms of structure and localization, focal adhesions formed in a three-dimensional (3D) setting differ from those in a two-dimensional (2D) setting^5^,^6^.

The details of the mechanical interactions between a cell and the ECM are important to the understanding of cell migration behavior. Such interactions have been suggested to be tightly linked to cellular mechanosensing activities that play roles in regulating lamellipodial protrusion and the formation of focal adhesions^7^,^8^. Moreover, the mechanosensing activities of ECs affect their sensitivity to vascular endothelial growth factor (VEGF)^9^, thus potentially influencing their migratory directions. Such studies also have implications for biomedical science and clinical practice. It may be possible to differentiate physiological from pathological angiogenesis based on the ways in which the cells interact with the matrices in these two conditions that probably have different mechanical properties^10^,^11^. Studying the regulatory effects of integrin on cellular interactions with ECM proteins may also contribute to enhancing the clinical benefit of *α*v-integrin antagonists^12^.

Many studies have suggested that ECs secrete a wide spectrum of matrix metalloproteinases (MMPs) that influence matrix remodeling, but the mechanisms underlying these processes are still poorly understood. In^13^, immunofluorescent staining for membrane type 1-MMP, which may co-localize with the proteolytic forms of MMP2 and MMP9, showed that it appeared alongside a certain portion of the ECs’ membrane that had formed apparent filopodia. The potential localized effects of membrane type 1-MMP were not considered in the present work, which focused on an expanded area of up to 200 *μ*m × 200 *μ*m. The soluble species, including MMP1 and MMP2, might also exist to degrade the collagen fibers in relatively distant areas, thus leading to reduced efforts made by the ECs deforming those areas. However, the purpose of this work was to identify deformation patterns rather than resolve forces that would be heavily dependent on the mechanical properties of the ECM, which warrants the neglect of those MMPs’ effects herein.

The work reported here partially revealed how the tip and stalk cells mechanically interacted with the 3D ECM during angiogenesis. The study of these cells belonging to multicellular structures marks a point of departure from most existing studies that focused on a single cell migrating over a substrate. The reported differences between the roles of the tip and stalk cells during angiogenesis warrants separate characterization of their interaction behavior, the results of which revealed the stalk cells to exhibit fewer types of interaction with the ECM than the tip cells. In this work, we were the first, to our knowledge, to establish the spatiotemporal interaction behavior of the tip cells in the context of reshaping (or remodeling) the surrounding ECM combined with the evolution of the lamellipodia (and filopodia) over time. This behavior was described in detail based on the associated movement of a mass of beads bound to collagen fibers. An investigation into the lamellipodia with distinct processes of evolution was also performed to explore their differences in interacting with the ECM.

## Results

### Tip Cells Exhibited Spatial and Temporal Interactions with ECM

**Figure 1:**
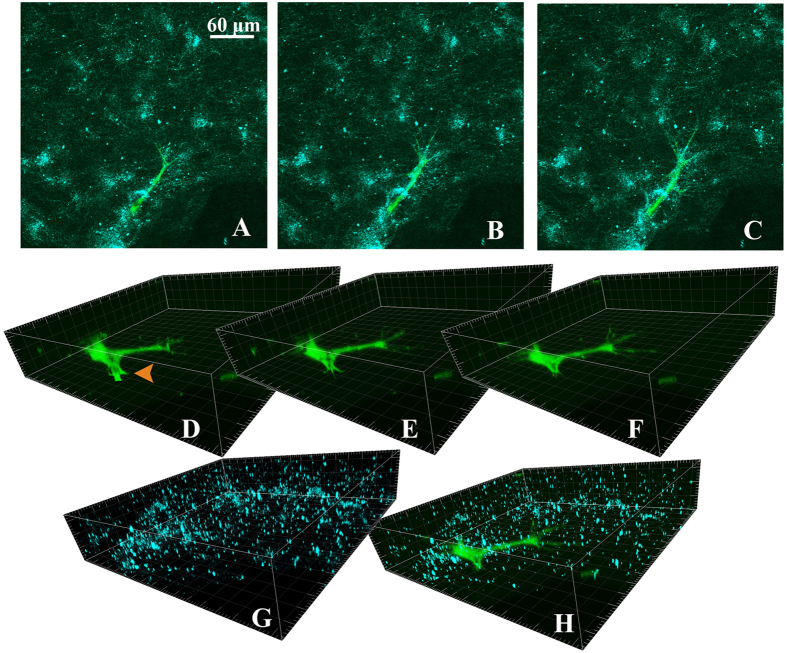
Images of a sprout and beads contained within the ECM. Immunofluorescence staining of the sprout with CMFDA was performed nearly 1 hour before imaging. To reduce the potential toxicity of the dyes, the cell channel and the opposite media channel of the devices were rinsed by delivering the media with the VEGF gradient into both channels after 30 minutes of staining had been completed. The images were acquired using an Olympus FV1200 confocal microscope with a 60× oil immersion objective. (**A**–**C**) collectively show a sprout (green) invading the ECM (with collagen fibers and beads displayed in cyan.) (**D**,**E**) solely show 3D views of the sprout in which the lamellipodia experiencing retraction were marked with an arrowhead. (**G**) solely shows the beads introduced in the ECM. A combined image is shown in (**H**).

Figure 1 presents confocal microscopy images showing the frontal part of a sprout immersed in the ECM. The details of the sprout’s structure can be seen in panels D–F, and the beads embedded in the ECM are shown in panels G and H.

The neighboring regions of the observed tip cells, particularly those close to their filopodia, were constantly deformed either toward or outward from the cells. Such opposing movements could be mediated simultaneously by the same tip cell in the different filopodial regions or even in the same region. The deformed regions almost fully recovered after the completion of the interactions or the existence of an opposite deformation in the same region.

**Figure 2:**
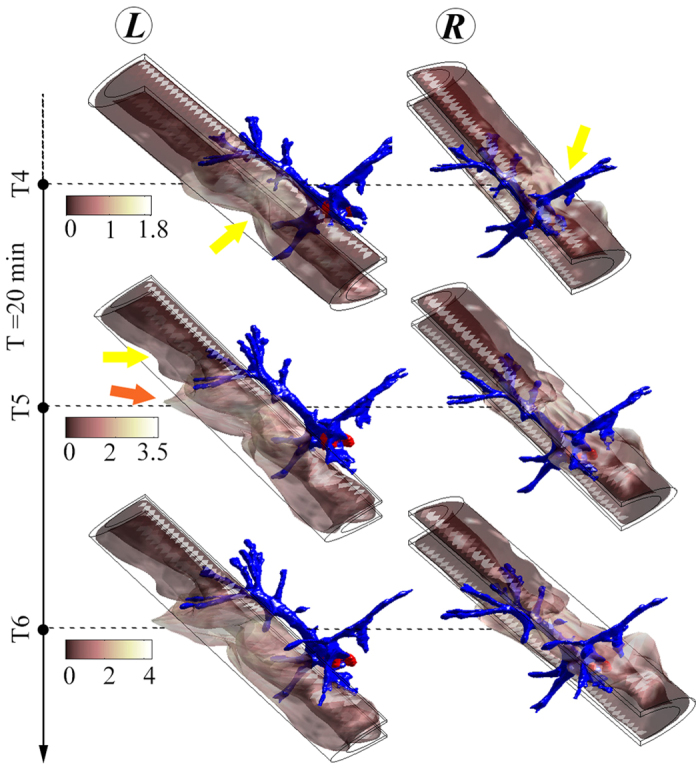
3D rendering of the ECM interacting with filopodia (blue) of a sprout tip (whose nucleus was indicated in red). Four time points (T3-T6) were involved, covering the period of one hour. The profile of the sprout tip was extracted with Imaris and reconstructed with MATLAB. The ECM shown here is a 10 *μ*m-thick cylindrical layer wrapping the sprout tip. The outside diameter of the layer is 70 *μ*m, whereas the length is 250 *μ*m. The cylinder was further separated into two parts to fully expose the structure of the filopodia. The outline of the undeformed ECM is shown with black lines. The deformation that appeared before the start of this 1-hour investigation (i.e., *prior to* the time point T3) is not reflected in these images. The significant deformations reflecting the pulling and pushing behavior of filopodia were marked with arrows. The actual levels of the deformation (shown with colorbar) were multiplied by a factor of 15 during image generation to allow better visualization. unit: *μ*m.

Figure 2 shows the typical interactions between the ECM and the front of a tip cell, as observed for about 1 hour. The identified behavior in these scenarios regularly occurred with the evolution of the filopodia for the observed tip cells. In this figure, three sets of results are presented with each involving a time interval of 20 minutes. The deformation that appeared before the start of this 1-hour investigation is not reflected in these images. The 10 *μ*m-thick cylindrical layer of the ECM was extracted to elucidate its shape change due to the cellular interaction behavior. The ECM cylinder was separated into two halves to fully expose the tip cell’s filopodia (in blue). The outline of the undeformed ECM is shown with black lines. For the first 20 minutes, a pull (marked by yellow arrow) of the ECM was observed on both sides of the branches that were more prominently formed than those at the very tip of the blue structure. As the tip (of the blue structure) developed during the second 20 minutes both a pull (yellow arrow) and a push (orange arrow) of the ECM in its neighboring region were pronounced. It is likely that the filopodia therein initiated the pull on the ECM during this period; meanwhile, their extension contributed to the push in the same region. This situation persisted for another 20 minutes. The observed pull on the right side of the ECM continued over the whole period, leading to a total of about 4 *μ*m shrinkage of the ECM.

During the imaging period, the filopodia were repeatedly observed to actively pull the ECM toward themselves, as well as push on it while protruding, as shown in Supplementary Figure S1, which constitutes continued analysis of the sprout tip shown in Fig. 2 for a period of time beginning 1 hour after the period covered by Fig. 2. The growth of the sprout tip over time is shown in Supplementary Figure S2, in addition to the extension rates of its different filopodia, which ranged from 1.5 *μ*m/h to 45 *μ*m/h, thus covering the values reported in^14^, another study investigating EC growth in collagen matrices.

### Tip Cell-ECM Interactions Comprised Four Basic Behavior Types

**Figure 3:**
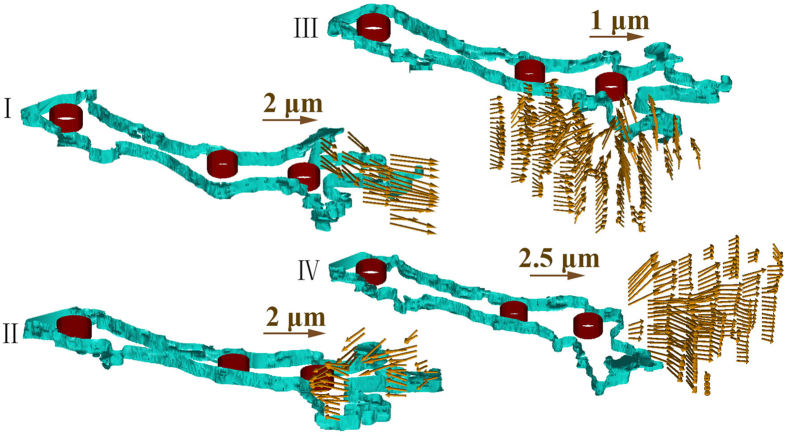
Four identified types of tip cell-ECM interaction behavior. *I*: “protrusion-related push”. *II*: “retraction-related push”. *III*: “pull”. *IV*: “release”. The shown vector fields were the results of tracing the beads in a 3D space with the method of DVC in^52^. The 3D outlines of the sprout and nucleus of each EC involved, in cyan and dark red, respectively, were built with their boundary contours extracted at the different depths using the open-source MATLAB software Sliceomatic^57^. The arrows were plotted using the MATLAB code provided by^58^. The “pull” type behavior was linked to a radial movement of beads in neighboring regions of a protruding lamellipodium with their directions of movement pointing toward the lamellipodium from all directions. The “release” type was particularly associated with a retracting lamellipodium, the surrounding beads of which moved radially away from it. For the “protrusion-related push”, the beads that were located above and very close to a protruding lamellipodium moved along with the protrusion. For the “retraction-related push,” the beads at the back of a retracting lamellipodium were pushed away approximately in direction of the lamellipodial retraction.

The observed tip cells basically involved four types of interaction behavior: “pull,” “release,” “protrusion-related push,” and “retraction-related push.” Each type was characterized by the identified features of the associated bead movement, as shown in Fig. 3.

**Figure 4:**
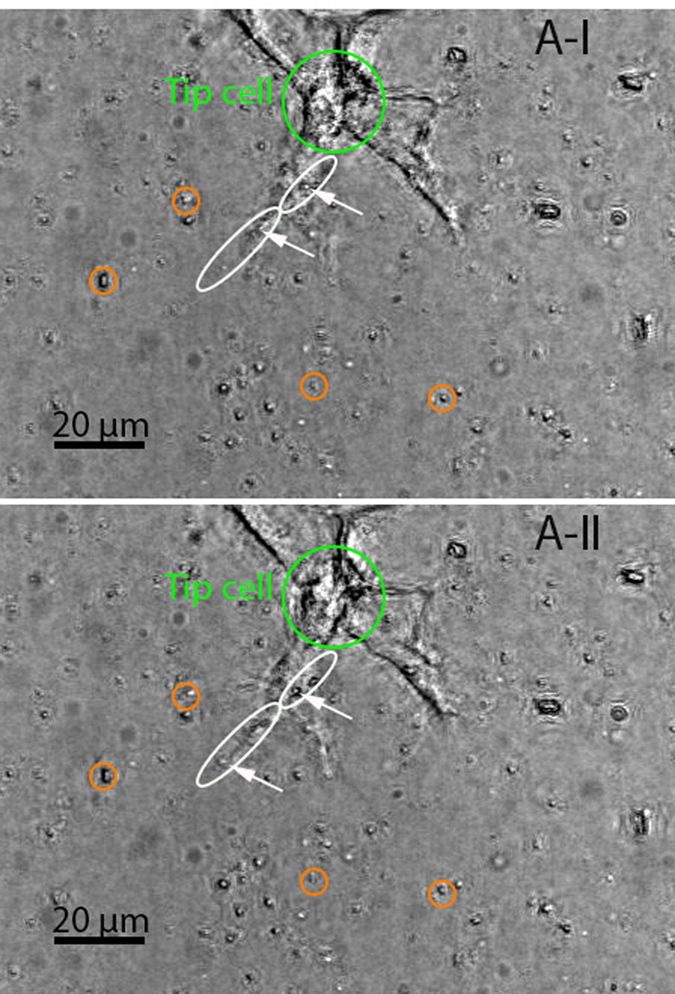
Experimental observations of the “pull” and “protrusion-related push” behavior of the tip cell (in green circle). These two in-focus images were generated with the two stacks of bright-field images (with a z-step size of 0.75 *μ*m) that were sequentially acquired with a time interval of 15 minutes. The beads that had the observable movement pattern associated with the “pull” type behavior were highlighted with orange circles in both images whose centers were coincident with the locations of those beads in image A-I. They were pulled toward the neighboring protruding lamellipodium, as manifested in image A-II by the deviatioin of their positions from the centers of the circles containing them. It should be noted that the highlighted beads were not in the same plane (which was observed *prior to* focus stacking). The beads (shown by arrows) linked to the “protrusion-related push” type behavior had a trajectory along the major axis of their relevant ellipse, almost consistent with the protruding direction of the associated lamellipodia.

The “pull” type behavior was linked to a radial movement of beads in neighboring regions of a protruding lamellipodium with their directions of movement pointing toward the lamellipodium from all directions. Those beads proximal to the lamellipodium were more profoundly displaced compared with the others. Direct observation of the bright-field images provided a clue to the pattern of the “pull” type behavior identified by tracing beads in a 3D space using the DVC technique. Figure 4 shows a pair of consecutive focused bright-field images generated with 85 slices (with a z-step size of 0.75 *μ*m). The beads that had the observable movement pattern associated with the “pull” type behavior were highlighted with orange circles in both images. The circles were placed in a way that showed the consistency of their centers with the locations of those beads *prior to* the movement (shown in image A-I). The changes in the positions of those beads relevant to the circles containing them, as can be observed in image A-II (that was acquired 15 minutes after A-I), manifested that they were moving slightly toward the neighboring lamellipodium during the time that elapsed between the two images. It should be noted that the highlighted beads were not in the same plane *prior to* focus stacking, suggesting that the “pull” type behavior could be out-of-plane action rather than the in-plane action reported by most existing studies on cells cultured on substrates. The estimated area of influence for a tip or stalk cell is described later. The collagen fibers remodeled as a consequence of this “pull” behavior were further investigated via collagen fiber-tracking, the results of which are shown in Supplementary Figure S4A and S4B. The “pull” behavior resulted in a densified layer of collagen fibers surrounding the lamellipodium. This caused a sparsity of collagen fibers in relatively distant areas (i.e., hundreds of microns away from the lamellipodium), with the lowest fiber concentration emerging in the region in front of the lamellipodium. Along with the repositioning effect, the “pull” behavior may have also led to the reorientation of the collagen fibers. The collagen fibers near the tip of the lamellipodium were usually oriented along its long-axis direction. This finding supports the suggestion in^15^,^16^ that the cell-mediated compaction of collagen fiber networks would result in fiber alignments, probably serving as “contact guidance” for cell migration^17^,^18^.

**Figure 5:**
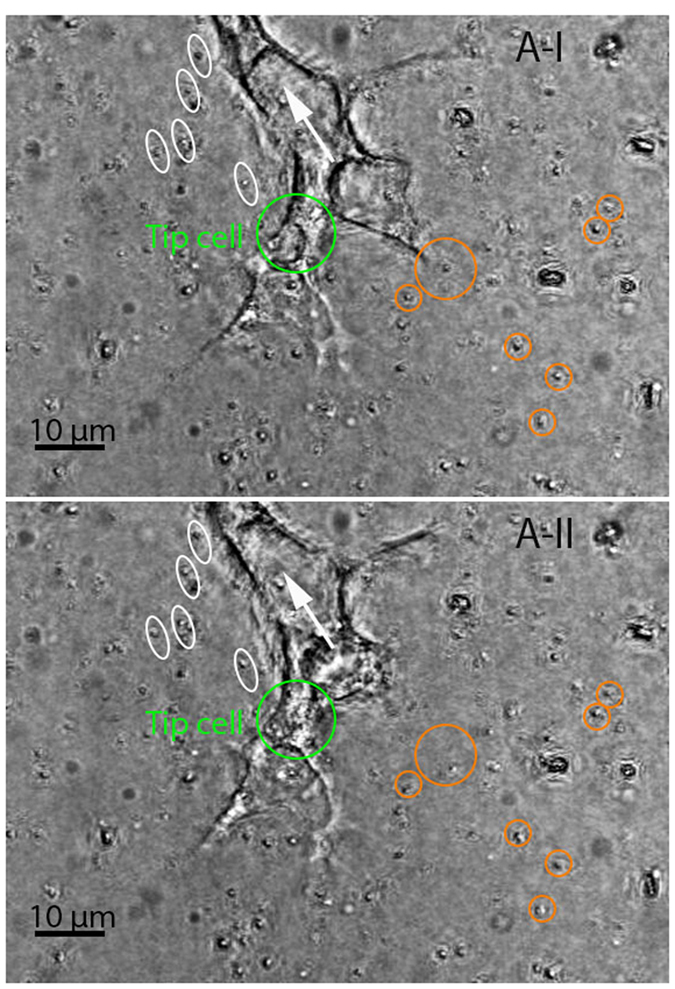
Experimental observations of the “retraction-related push” and “release” behavior of the tip cell (in green ellipse). Images were produced the same way that those of Fig. 4 were generated. For the “retraction-related push,” the involved beads (in ellipses) located at the back of the retracting lamellipodium were pushed away from the lamellipodium in a direction that was consistent with that of the major axes of those ellipses, which approximates the lamellipodium’s retraction direction (shown by an arrow). The beads (in orange circles) linked to the “release” behavior of the retracting lamellipodium were radially displaced away from the lamellipodium. Among them, those (e.g., the one inside the largest circle) closer to the lamellipodium were more significantly displaced than the others in the same group. It should be noted that the highlighted beads were not in the same plane (which was observable *prior to* focus stacking), indicating the presence of out-of-plane action.

The “protrusion-related push” usually appeared in association with the “pull” behavior. The beads located above and very close to a protruding lamellipodium moved along with the protrusion, but with a much smaller magnitude than that of the associated protrusion. This motion was observed in the two beads shown by two arrows in image A-I of Fig. 4. By comparing images A-I and A-II, it can be seen that these two beads were displaced along the major axis of the ellipses surrounding them, which was highly consistent with the protruding direction of the associated lamellipodium. It is likely that this type of push was only an “additional product” of lamellipodial protrusion and thus had no effect on cell migration behavior. A similar movement may have also occurred in the beads underneath the protruding lamellipodium. In addition, a “retraction-related push” behavior existed in which the beads at the back of a retracting lamellipodium were pushed away in the direction of lamellipodial retraction, as seen in those surrounded by ellipses in image A-I of Fig. 5. The ellipses were oriented in a way that showed the consistency of their major axis with the relevant bead trajectory formed during the time between acquisition of the two images. The alignment of those ellipses was observed to be largely coincident with the retraction direction (marked by an arrow) of the neighboring lamellipodium.

The “release” type behavior was usually observed in retracting lamellipodia, coupling to the movement of the surrounding beads away from them in the radial direction, as illustrated by those highlighted with circles in image A-I of Fig. 5. Similar to the identified “pull” type, the “release” type featured out-of-plane movement indicated by the results from the DVC analysis. This release action might have been caused by the breakage of lamellipodium-matrix bonds with the retraction of the lamellipodium. Once released, the collagen fibers were able to return to their original positions due to their inherent viscoelastic properties. A clear path of recovery can be observed in Supplementary Figure S4C and S4D. The consequence of this “release” action was in agreement with the record of second harmonic generation of collagen linked to retraction of the tip cell filopodia in^19^, showing the decompaction of collagen fibers that were previously recruited by the filopodia.

### Coupling of Tip Cell-ECM Interactions with Lamellipodia Evolution

**Figure 6:**
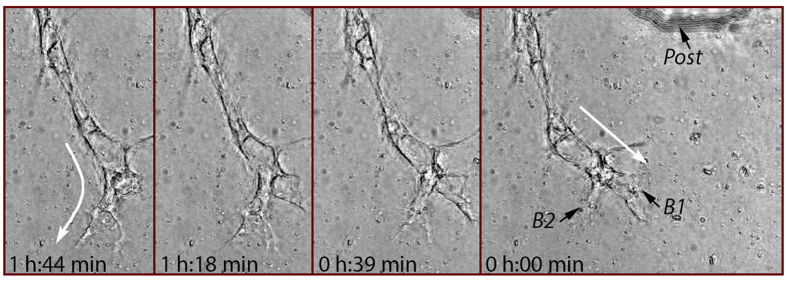
Sprout elongation with both lamellipodial protrusion and retraction over a period of nearly 2 hours. The tip cell heading (marked by arrows) varied with the different evolving processes of tip cell lamellipodium branches B1 and B2. The sprout changed its heading from the long-axis direction of B1 that was undergoing retraction to that of B2 continually protruding.

As the tip cell was invading the ECM, the involvement of the identified interaction behavior and the persistence of each associated behavior were heavily linked to the evolution of its lamellipodia. Figure 6 shows a representative sprout shifting its heading over a 2-hour period when the two lamellipodium branches (i.e., B1 and B2) of its tip cell were evolving quite differently. The tip cell initially headed in a direction (marked by an arrow in the rightmost image) that was almost consistent with the approximate long-axis direction of B1. At this initial stage, B1 was more prominently formed than B2. About 1 hour later, B1 had retracted significantly, whereas B2 continued to protrude with a few of filopodia-like cytoplasmic projections extending outward. This was associated with the shifting of the tip cell’s heading to the approximate long-axis direction of B2 (which concurred with the finding in the study^19^), as indicated by a curved arrow in the leftmost image.

**Figure 7:**
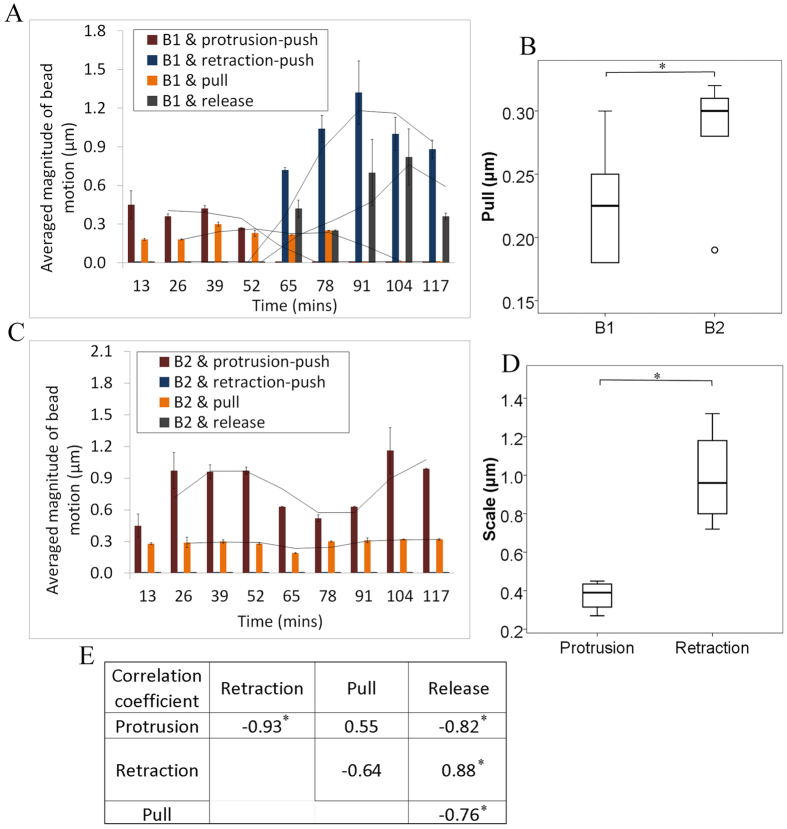
The 2-hour dynamics of interaction with the ECM of the two tip-cell lamellipodium branches, B1 and B2, which exhibited distinct morphological changes (shown in Fig. 6) during this period. Panels (**A**,**C**) reveal the involvement of the four interaction behavior types at each time point for B1 and B2, respectively. It is important to note that only the deformation appearing between two adjacent time points is reflected in the result for the latter time point, meaning that deformation did not accumulate over time. (**B**) shows a comparison in the pull scale between B1 and B2. The significance of the difference was evaluated with an independent samples t-test in IBM SPSS Statistics 22 (*P* < 0.05). (**D**) shows a comparison of the scales of both protrusion and retraction for B1. (**E**) lists the computed Pearson correlation coefficients for these six pairs of interaction behavior types.

These two lamellipodium branches exhibited differentiated interaction behavior with distinct processes of evolution, as shown in Fig. 7A for B1 and 7C for B2. The method of quantitatively analyzing such behavior is illustrated in Supplementary Figure S7. B1 exclusively underwent a transition from the “protrusion-related push” to the “retraction-related push” and from the “pull” to the “release” behavior. Moreover, the average “retraction-related push” scale of about 1.0 *μ*m was significantly higher than the average “protrusion-related push” scale of about 0.4 *μ*m 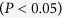, as revealed by the independent samples t-test. *Prior to* the retraction, the B1 exerted an average “pull” of about 0.23 *μ*m in terms of bead movement, which was significantly lower 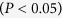 than the 0.31 *μ*m of the “pull” of the continually protruding B2 branch. As indicated in Fig. 7E, certain degrees of correlation were present within these interaction behavior types. There was a moderate positive correlation between the “protrusion-related push” and the “pull” with a 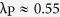. In contrast, the positive correlation between the “retraction-related push” and the “release” was much stronger, with a 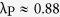. Along with these positive correlations, negative correlations existed within the four pairs; namely, the “protrusion-related push” and “retraction-related push,” the “protrusion-related push” and “release,” the “retraction-related push” and “pull,” and the “pull” and “release” behavior types.

### Stalk Cells Exhibited Less-complex Interaction Behavior Than Tip Cells

**Figure 8:**
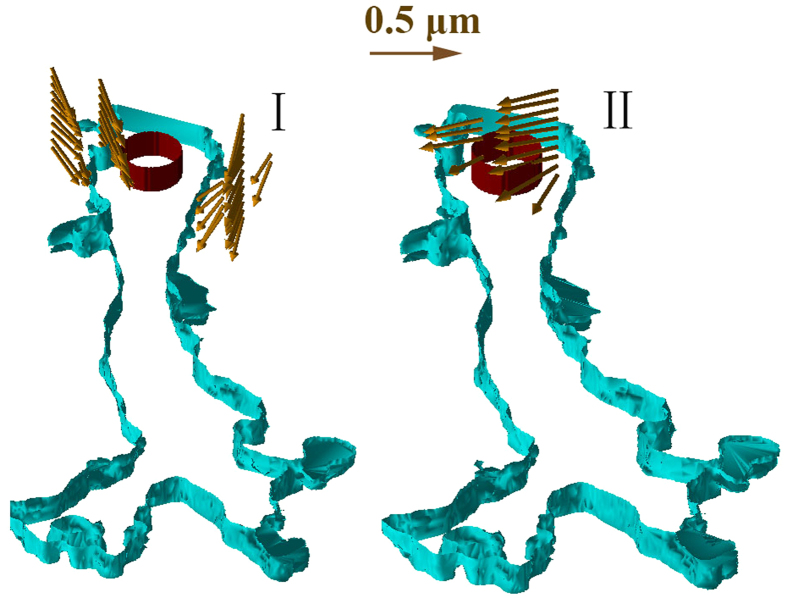
Two identified types of stalk cell-ECM interaction behavior. *I*: “pull”. *II*: “migration-related push”. Similar to the tip cells, the stalk cell (in dark red) also manifested a “pulling” behavior, driving the radial movement of the surrounding beads toward itself. The “migration-related push” was also observed, and involved the beads that moved along with the “shuffling” (i.e., backward and forward movement) of the stalk cell.

**Figure 9:**
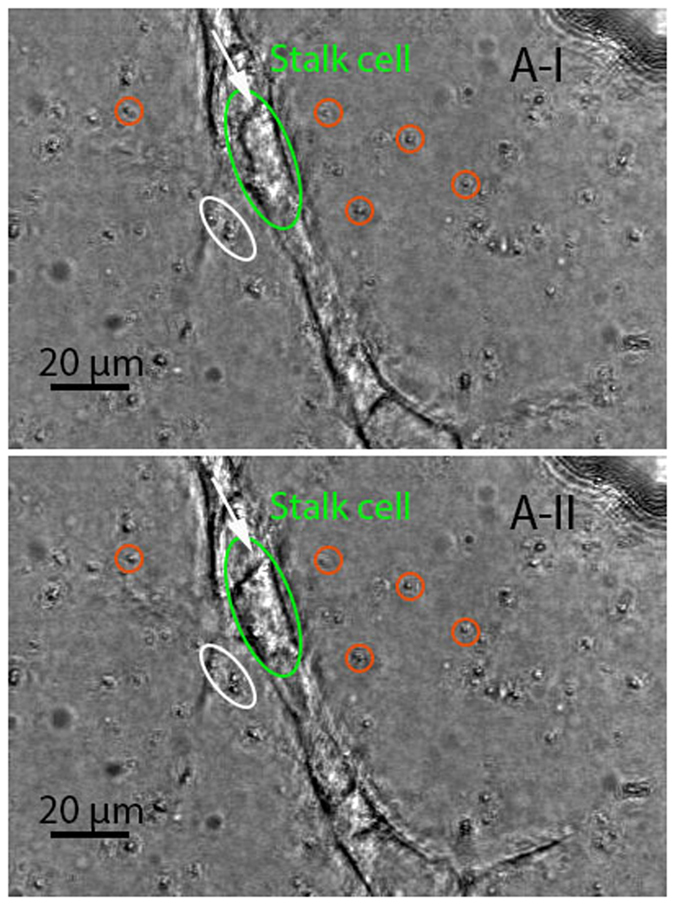
Experimental observations of the bead movement manifesting the interactions between a stalk cell (in green ellipse) and the ECM. These two consecutive (T = 15 minutes) focused bright-field images were generated with 85 slices (with a step size of 0.75 um). The stalk cell migrated along the longitudinal axis of the lumen during the time lapsed between the two images. The beads (in ellipse) close to the stalk cell featured a similar movement to that of the cell, suggesting that there was a possibility that the stalk cell migration passively displaced the nearby region, called “migration-related push” herein. Those highlighted with circles slightly moved toward the stalk cell, showing the same “pull” behavior as that of the tip cells, but less pronounced.

We observed two main types of stalk cell-ECM interaction behavior; namely, “migration-related push” and “pull,” as demonstrated in Fig. 8. The “migration-related push” was manifested by the beads moving along with the “shuffling” (i.e., backward and forward movement) of the stalk cell that they were very close to. An illustration of this movement is shown in consecutive images A-I and A-II in Fig. 9, covering a short period of about 15 minutes. During this period, the stalk cell (in green ellipse) migrated in the direction shown by an arrow, accompanied by the similar movement of the two beads (in white ellipse) proximal to the cell. The “shuffling” movement of stalk cells has been reported as part of sprout elongation in^20^,^21^. The presence of such a movement for stalk cells breaks a simple assumption in^22^,^23^ about the mechanism underlying sprout elongation, called a “navigate-pull-proliferate” process, in which tip cells in sprouts navigate as leaders and regularly impose pulling forces on the adjacent stalk cell segments, triggering stalk cell proliferations that dominantly drive sprout elongation.

The stalk cells also manifested a “pulling” behavior, driving the radial movement of the surrounding beads toward themselves but to a lesser extent than the tip cells. An illustration can be seen in those highlighted with circles in image A-I of Fig. 9. However, the influence of such behavior over the stalk cells tended to be more localized relative to that for the tip cells. Our observation of this behavior for the stalk cells further verified the existence of their migratory behavior by providing evidence of the presence of a contractile force that probably steered the migratory behavior in a similar manner exhibited by other types of cells.

### Stalk Cells Influenced More Confined Areas Than Tip Cells

**Figure 10:**
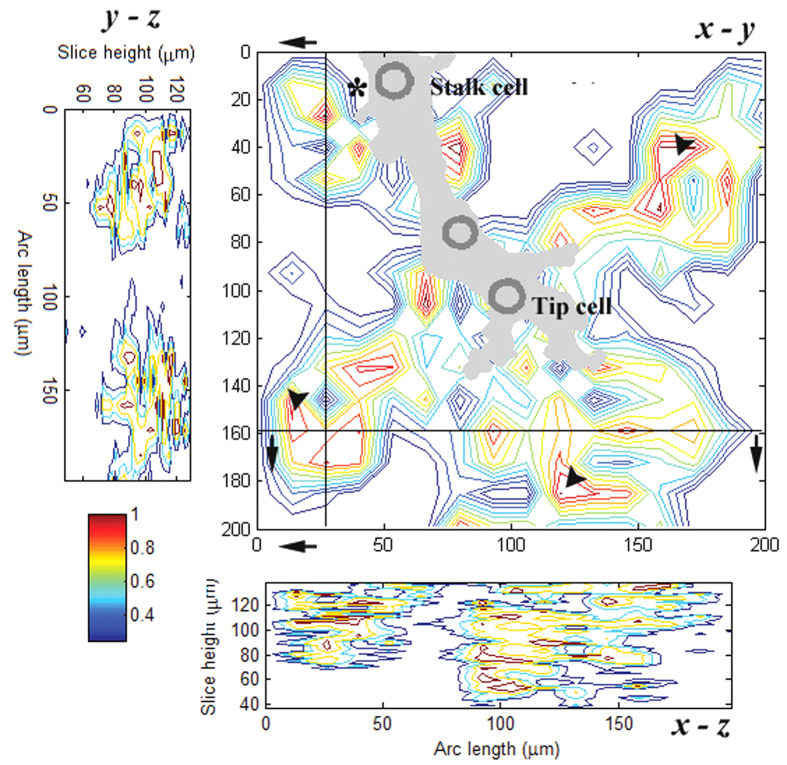
The area of influence for a sprout involving a tip cell and two stalk cells. The magnitude of deformation in a region was measured as an indicator of the manipulation of this region by the ECs involved. The *z*-directional effect is shown in the cross-sectional views, i.e. the 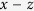 plane at 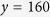 *μ*m and the 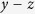 plane at *x* = 25 *μ*m. The arrowheads mark the most active regions that emerged in the areas 50–60 *μ*m away from the tip cell lamellipodia in the radial direction.

In addition to being less complicated, the interactions between the stalk cells and the ECMs covered more confined regions than those occurring between the tip cells and the ECMs. Figure 10 illustrates the regions affected by the stalk and tip cells of a typical sprout in three dimensions. It was constructed based on the magnitudes of the 3D bead displacements. The cross-sectional views at the two positions (*y* = 160 *μ*m and *x* = 25 *μ*m, which are indicated by the two black lines on the 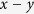 plane) are also displayed.

On the 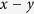 plane (at *z* = 105 *μ*m), it is apparent that the stalk cell (marked by the “*” symbol) only affected the region near it, approximately less than 45 *μ*m away. In contrast, the tip cell’s influence spread over regions up to 70 *μ*m away. Furthermore, the shape of these tip cell-related regions as a whole resembled that of the sprout tip. The most active regions (marked by the arrowheads) were observed to emerge at the locations distant from the tip cell lamellipodia instead of appearing at those very near to the lamellipodia. One possible reason for the differences in the sizes of the affected regions between the tip and stalk cells was that stalk cells usually do not form long filopodia. However, these two types of ECs showed negligible differences in displacing the ECM in the *z* direction. On the 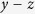 plane, the depths of influence imposed by the stalk and tip cells were basically the same, i.e., nearly 60 *μ*m. By only considering the tip cell, the *z*-directional influences of its different lamellipodia might not necessarily be the same, as shown in the bottom image of Fig. 10.

## Discussion

Using beads as a tool to probe cell-ECM interactions allows the analysis of the deformation field throughout the entire ECM while minimally interfering with cell activities. This is based on (i) the average gap between two adjacent beads (nearly 14 *μ*m over a 227 × 105-*μ*m region in the image plane that best represented the sprout center) was sufficient to allow filopodia (routinely in a diameter ranging from 0.1 *μ*m to 0.3 *μ*m^24^,^25^) to freely protrude and swerve within the bead-containing ECM and (ii) the change in ECM stiffness produced by the beads at the concentration used (i.e., 0.075 mg/ml) was negligible. The latter concurred with our previous studys finding that a 5% change in ECM stiffness was associated with the addition to the ECM of 0.1 mg/ml of beads of the same size as those used in the current study^26^.

Our observation of the centripetally distributed bead displacement vectors pointing inward toward the tip cell from all directions, an indicator of the out-of-plane pull action exerted by the tip cell, extends the reported 2D pull behavior of the cells cultured on substrates in a number of studies (e.g.^3^,^7^). Such radial movement is in line with the finding reported in^27^ concerning the fanlike reorientation of collagen fibrils near the tips of early-stage sprouts across multiple image slices, which formed from the microvessel fragments that were cultured within the collagen gels. This collagen fibril realignment extended to distant areas of the microvessel sprout tips, a phenomenon also manifested in our investigation of the area of influence of a typical sprout. The identified push exerted by the tip cell on the ECM in conjunction with the lamellipodial (and filopodial) protrusion, however, contradicts the suggestion in^28^ that filopodia usually protrude along collagen fibers with only negligible pushing forces imposed on the fibers. The moderate positive correlation 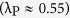 between the contraction-related pull and the protrusion-related push indicates that a balance between the protrusive and contractile forces is probably required for cells migrating in 3D matrices, and not just for those migrating over substrates, as suggested in^29^,^30^,^31^. The retraction-related push on the matrix was probably due to the failure of the lamellipodium to form stable attachments with the ECM. Multiple factors were thought attributable to such unstable attachments, such as receptor signaling, myosin-based contraction or ECM adhesiveness^32^.

The changes in the headings of the tip cells were usually observed to link with the retraction of selected lamellipodia (and filopodia) and the continuing protrusion of most of the remaining ones. Furthermore, the heading was more likely to align with the long-axis direction of the continually protruding lamellipodium that had formed mature contacts with the ECM, as suggested in^32^,^33^. The selection of the lamellipodia to protrude or retract was possibly based on cellular sensing of ECM rigidity with the regulation of Rho kinase-mediated myosin II activity^34^. The lamellipodium being retracted involved distinctly different dynamics of interaction with the ECM compared to those associated with the lamellipodium being protruded. An apparent difference existed in their pulling scales. The retracting lamellipodium pulled on the ECM to a significantly smaller degree than the continually protruding one, which in turn determined their diverse morphologies, according to the “motor clutch” model developed in^35^,^36^. Compared with the retracted lamellipodium, the continually protruded one exerted a larger pull on the ECM that resulted in a higher loading force on the ECM (which can be understood based on Hooke’s law) and thus experienced greater resistance in the myosin-driven retrograde flow and produced a more profound protrusion at the end of the F-actin bundle.

The issue of how stalk cells support sprout elongation is still unclear. A general assumption is that a sprout elongates as the stalk cells proliferate (triggered by VEGF-A)^37^,^38^ or as they are pulled by the tip cell^22^; however, “shuffling”, in which backward and forward movements of the stalk cells push the sprout forward has also been suggested^20^,^21^. Such movements were thought to cause a conversion of cell phenotype^20^, but whether the velocity is a major factor in this conversion remains unknown. Consistent with the latter of these two opinions, our finding of the “migration-related push” behavior of the stalk cells along the migratory directions hints at the migration-driven stretches of lumens where the stalk cells were resting. In addition to the “migration-related push”, the “pull” was also found for the stalk cells, which may be coupled to their “shuffling” movements or cytoskeleton organization that has been reported to be crucial to cell survival^39^,^40^. The mechanical influences of the stalk cells via the pull actions were not as extensive as those of the tip cells, due to the absence of long filopodia in stalk cells.

The behavior of tip and stalk cells belonging to the same sprout when interacting with the ECM collectively repositioned and reoriented the fibers to form a well-organized ECM structure. Through its out-of-plane pulling actions, the tip cell lamellipodia gathered collagen fibers from faraway regions towards themselves, resulting a heterogeneous spatial distribution of collagen fibers in the matrix. How a matrix with such a structure facilitates the functions of the sprout is unclear. We suspect that the densification of collagen fibers occurring in the regions in the vicinity of the sprout may help to support it physically, and the resulting sparseness of collagen fiber distribution at relatively remote regions may reduce its efforts to invade those regions later. Such a modification of the ECM structure seems almost reversible because we observed that when the release action was performed by the retracting lamellipodium, large amounts of collagen fibers clustering in the region of this lamellipodium returned to their original positions. In conjunction with their repositioning, the collagen fibers were usually rearranged. For example, those distributed within the anterior region of lamellipodia were more likely to be aligned to orient in their long-axis directions.

The sample size used in this study was heavily restricted by the designated rules for selecting the sprouts for imaging, including (i) for each set of experiments, a very limited number of devices could be captured consecutively due to concern over the consistency of maturity of the sprouts to be analyzed, and (ii) for each device, only one selected sprout could be observed due to the fixed microscopy stage used to avoid unseen consequences of position drift. Although a limited number of samples were used, they were properly representative of those under the same experimental conditions. Thus, the in-depth study we performed may provide some insights into the general behavior of these sprouts. We expect that in the future, the preliminary results obtained to date could be enhanced through extensive studies combining the techniques of tracking molecular signalling pathways such as Rac, Cdc42, talin, vinculin and VEGFR-2. In addition, it would be interesting to know whether the varying mechanical properties of the 3D matrices would affect the cellular activities in the same ways as the 2D matrices.

Our deployment of an *in vitro* assay involving ECs alone, which possessed the advantages of being reproducible and quantifiable, allowed for preliminary screening for EC-ECM interactions in the early stage of sprouting angiogenesis in view of potential variations in the interaction patterns among different *in vitro* angiogenesis assays, and between *in vitro* and *in vivo* assays. The results could potentially be extended by applying the methods of bead and collagen fiber tracking to *in vitro* co-culture assays (involving ECs and supporting cells such as smooth muscle cells, pericytes and fibroblasts) or even *in vivo* assays that trigger more “natural” EC behavior in angiogenesis. The supplementation of the basal medium with FBS for cell proliferation and attachment also can potentially negatively affect the generality of the results produced due to the ambiguity of the composition of the serum^41^. We selected the regions of the ECM that contained a sole sprout for imaging. Although this approach permitted a pure ECM deformation field to be extracted for the target, the results may not be fully representative of cases in which target sprouts are closely surrounded by other sprouts owing to potential changes in their interactions with the ECM resulting from competition with other sprouts for resources at the cellular level and alterations in ECM properties caused by the presence of other sprouts. The current study was limited to early-stage sprouts, but it would be desirable to also seek an understanding of the interplay between ECs and the ECM in the later stages of angiogenesis such as network formation, vascular remodeling and pruning, and stabilization. Despite the observed repeatability of the identified interactions of the ECs with the ECM, it would still be desirable in a future study to capture sprouts over a longer period during which they would likely become less motile and change their interaction behavior to some degree.

## Materials and Methods

### Microfluidic Device

**Figure 11:**
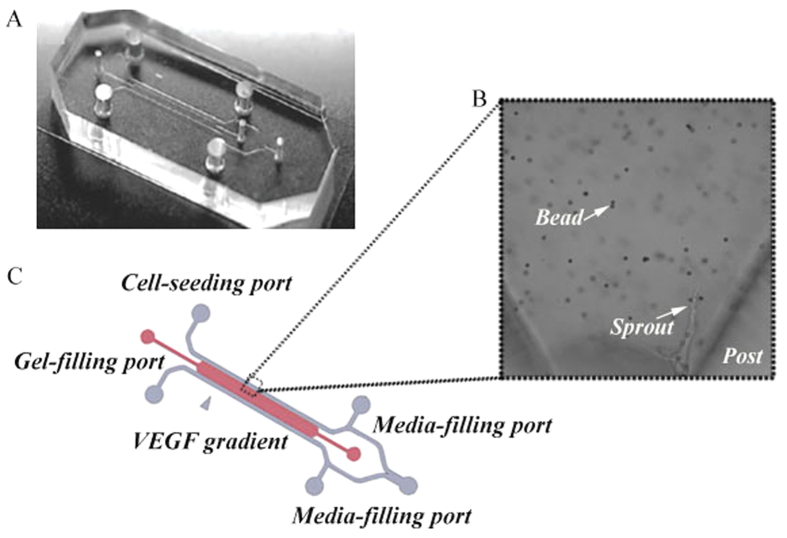
The microfluidic device. (**A**) Photograph of the actual device. (**B**) Microscopic view with a 60× oil immersion objective. (**C**) Design of the inner channels and inlets.

The design of microfluidic devices described in^42^ was adopted. Inlets were configured to allow collagen solution, media, and cell solution to be delivered to their respective channels (shown in Fig. 11C). The devices were fabricated from polydimethylsiloxane (PDMS - Dow Corning Sylgard 184) using standard soft lithography and plasma bonded to glass cover slides. The devices were transferred to a beaker with sterilized water for autoclaving and then dried in an oven at 80 °C prior to collagen injection.

### Preparation of ECM

The collagen solution was prepared on ice with 2.5 mg/ml Rat Tail Collagen Type I (BD Bioscience), sodium hydroxide, and 10X phosphate buffered saline. The solution of beads (D ≈1.5 *μ*m, BM551 from Bangs Laboratory) at a concentration of 0.075 mg/ml was mixed with the collagen solution. The beads were coated with streptavidin that contains an Arg-Tyr-Asp (RYD) amino acid sequence that mimics the Arg-Gly-Asp (RGD) receptor domain of fibronectin^43^, allowing tight binding between the collagen fibers and the beads^44^. The mixture of collagen and beads was thoroughly vortexed for 2 minutes in an ice bath until a homogeneous solution was formed. A volume of 20 *μl* of the mixture was pipetted into the gel-filling port. The gel-filled devices were then checked under microscopy to ensure that no air bubbles had been introduced in the process and that no leakage of the mixture into other channels had occurred. For gelation, the devices were placed in an incubator at 37 °C and 5% CO_2_. The local stiffness of polymerized gel with a similar concentration of collagen to that used in these experiments has been tested to 33.59 Pa in atomic force microscopy indentation experiments^45^. It is important to note that for soft biological material, the stiffness at the macro-scale level may be several thousands of times higher than that at the micro-scale level^46^.

### Cell Culture and Seeding

Human microvascular ECs (HMVECs, Lonza, Walkersville, MD) were cultured in endothelial growth medium-2MV (2% serum, Lonza) with the media changed every 2 days. The HMVECs were passaged once from passage 6–7 using EDTA solution (Gibco, Grand Island, NY), and then 2.5 × 10^6^ cells/mL of HMVECs were delivered to the cell-seeding channel in the microfluidic device by introducing a minor flow of the cell solution into the channel. Cells were allowed to grow and form a confluent cell monolayer on the glass substrate and the collagen wall before the start of the experiments. After being starved for 2 hours, HMVECs were treated with VEGF at 20 ng/mL in the cell-seeding channel and at 40 ng/mL in the media channel.

### Live Cell Microscopy

On days 5–6, time-lapse images of the microfluidic devices were acquired using an Olympus FV1200 confocal microscope with 60× oil immersion objective (NA = 1.42). We selected an oil immersion objective for imaging because (i) the ECM contained a network of collagen fibers accompanied by the beads bound to them, both of which had a high refractive index, i.e., 1.4–1.45 for the collagen fibers^47^,^48^ and 2.2–3 for the iron oxide beads^49^,^50^, and (ii) the collagen fibers at the interface between the ECM and coverslip were bound to the coverslip. A certain degree of geometric distortion can be observed in the bead images (e.g., their elongation in the z-axis), most likely because of the presence of a turbid medium, with the refractive index of the beads varying from that of the collagen fibers surrounding them.

It usually took about 8 hours to image each device. To ensure consistency in the maturity of the sprouts to be analyzed, no more than three devices could be captured consecutively within the same set of the experiments. Because the devices were prone to position drifts during relocalization of the microscope stage, which comprised the accuracy in tracking the cell-mediated activities even with the treatment of image alignment, the position of the microscope stage was fixed throughout the entire imaging period, allowing the capture of only one selected sprout for each device. The criteria for selecting the sprout to be imaged included (i) the sprout was exclusively occupying the microscopic field of view to avoid interference from other sprouts; (ii) the sprout was located far away from surrounding posts, which had much higher stiffness than the ECM; (iii) its tip cell clearly had multiple filopodia; and (iv) there was no sign of imminent breakage of the sprout.

*Prior to* imaging, staining of the nuclei and cell membrane of living ECs was performed with Hoechst (Invitrogen H1399) and CellTracker^TM^ CMFDA (5 chloro-methylfluorescein diacetate, Invitrogen C7025) for nearly 30 minutes. To reduce the potential toxicity of these dyes, the cell channel and the opposite media channel were rinsed once after staining by delivering media with the VEGF gradient into both channels. Fluorescent images were visualized using IMARIS 7.2 (Bitplane).

### Characterization of Bead Movement

The open-source code from MatPIV^51^ developed with particle image velocimetry (PIV) technique was used to track bead movement in the x–y plane (where the center of the target sprout was best represented) to compare the filopodial activities in that particular plane. 3D analysis of the deformation fields (or called Digital Volume Correlation (DVC) analysis) associated with both the tip and stalk cells was conducted with the help of an open source code^52^ capable of dealing with volumetric images in a 3D space. The robustness and precision (at the sub-pixel level) of this code have been tested, and are reported in^53^. Several alternative methods have also been developed to resolve 3D deformation fields throughout image volumes, such as the DVC technique in^54^ and the optical-flow analysis of microscopy image stacks in^55^. The vector fields produced using this code were further extended to a continuous solution (i.e., continuous over the entire volume of the ECM) to allow better visualization. Errors introduced by the possible drift of the device or the microscopy stage were eliminated by aligning the images using the area of the post in each bright-field image, that is, the stationary structure designed to prevent the collagen solution from leaking into the media channels. In-focus bright-field images were produced using the EDF plugin^56^ in ImageJ to show the involvement of beads associated with a wide range of depths in each type of the interaction behavior. The statistical analysis of the data was performed with IBM SPSS Statistics 22.

### 3D Rendering of Sprouts and ECM

The surfaces of the sprouts were rebuilt in Imaris using the CMFDA stain images and exported as VRML2 files. They were then converted to OBJ format with Blender 2.72b and read in MATLAB. The ECM was initially plotted and meshed in COMSOL Multiphysics 4.3, imported in Blender as STL files, and then reconstructed in MATLAB taking into consideration the deformation at all nodes. These deformations were interpolated from the values of those at the bead locations.

### Collagen Fiber Search

A technique was developed to recognize collagen fibers in the reflectance images and analyze their orientations. The reflectance images were thresholded to extract collagen fiber pixels *prior to* the search for collagen fibers. Salt and pepper noises yielded in the thresholding process were eliminated with a Wiener filter. For a collagen fiber pixel *p*, the possibility that there existed a left-titled (or right-titled) collagen fiber in a defined rectangular region where *p* appeared at its upper right corner (or upper left corner) was examined based on region intensity. The assumption that no collagen fibers existed at *p* would be made given a sufficiently low intensity in the region; otherwise, the presence of a collagen fiber would be determined. The orientation was given by the angle of the line connecting *p* and the central collagen fiber pixel in the region. The reconstruction of this collagen fiber was performed with knowledge of the locations of both *p* and the central pixel and its orientation.

An alternative method reported in^16^ was applied to verify our approach to quantify collagen fiber orientation. By using their method, a rectangular window was moved across the thresholded image. The defined moment tensor regarding the collagen-fiber pixels in the window was determined. The ratio of the elements in the eigenvector associated with the larger eigenvalue of the moment tensor yielded the collagen fiber orientation.

## Additional Information

**How to cite this article**: Du, Y. *et al.* Three-Dimensional Characterization of Mechanical Interactions between Endothelial Cells and Extracellular Matrix during Angiogenic Sprouting. *Sci. Rep.* **6**, 21362; doi: 10.1038/srep21362 (2016).

## Supplementary Material

Supplementary Information
