# Supplementary material for: Three-Dimensional Characterization of Mechanical Interactions between Endothelial Cells and Extracellular Matrix during Angiogenic Sprouting
Source: Sci Rep. 2016 Feb 23;6:21362. doi: 10.1038/srep21362 (PMC4763258; doi:10.1038/srep21362)
Supplement: Supplementary Information [file srep21362-s1.pdf]

Three-Dimensional Characterization of Mechanical Interactions between Endothelial Cells and Extracellular Matrix during Angiogenic Sprouting

YUE DU, SAHAN C. B. HERATH, QING-GUO WANG, DONG-AN WANG, H. HARRY ASADA, AND PETER C. Y. CHEN

Supplementary Figure S1	The repeatedly observed pull and push actions of the sprout tip
Supplementary Figure S2	Sprout growth and filopodial extension over the 6-hour period involving the time points T1-T18
Supplementary Figure S3	Results of the search for collagen fibers
Supplementary Figure S4	Reallocation of collagen fibers
Supplementary Figure S5	Reflectance intensity of collagen fibers in both the occupied and isolated regions
Supplementary Figure S6	Results of collagen fiber-orientation analysis of four regions
Supplementary Figure S7	Classification of bead movement

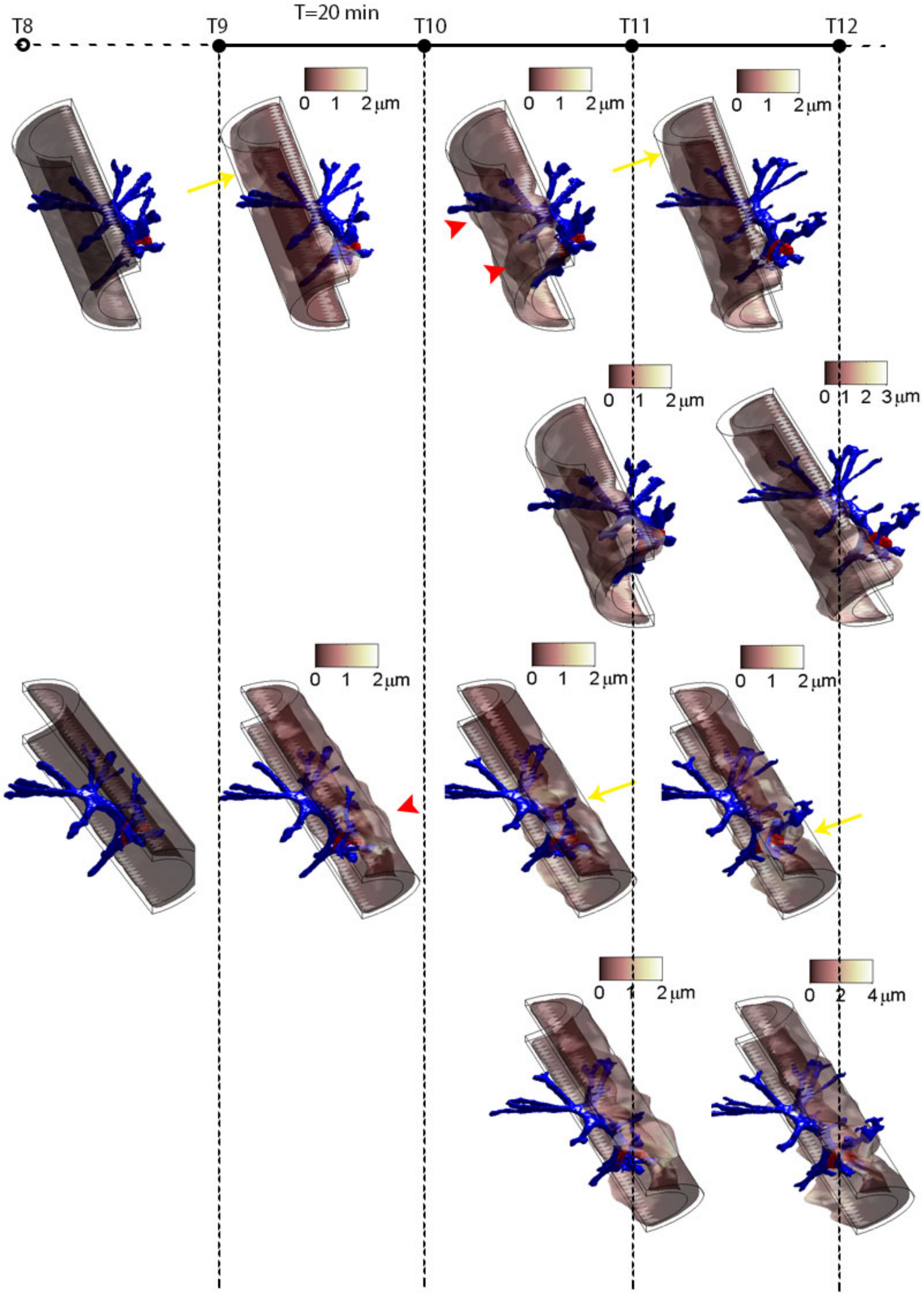
T=20 min
T8
T9
T10
T11
T12
0 1 2 μm
0 1 2 3 μm
0 2 4 μm

Figure S1. The repeatedly observed pull and push actions of the sprout tip. The result shown was obtained from continued analysis of the scenario in Figure 2 for a period of time beginning 1 hour after the period covered by Figure 2. Images in the first and third rows show the deformation appearing between two adjacent time points, which did not accumulate over time. Those in the second and fourth rows manifest the accumulated deformation emerging at the relevant time point. It should be noted that the deformation that occurred before the start of this 1-hour investigation was not included (shown in the leftmost images in the first and third rows). The significant deformations observed were marked by yellow arrows (pull) and orange arrow heads (push). The actual levels of the deformation (indicated by colorbar) were multiplied by a factor of 15 during image generation to allow better visualization. The time interval T = 20 min.

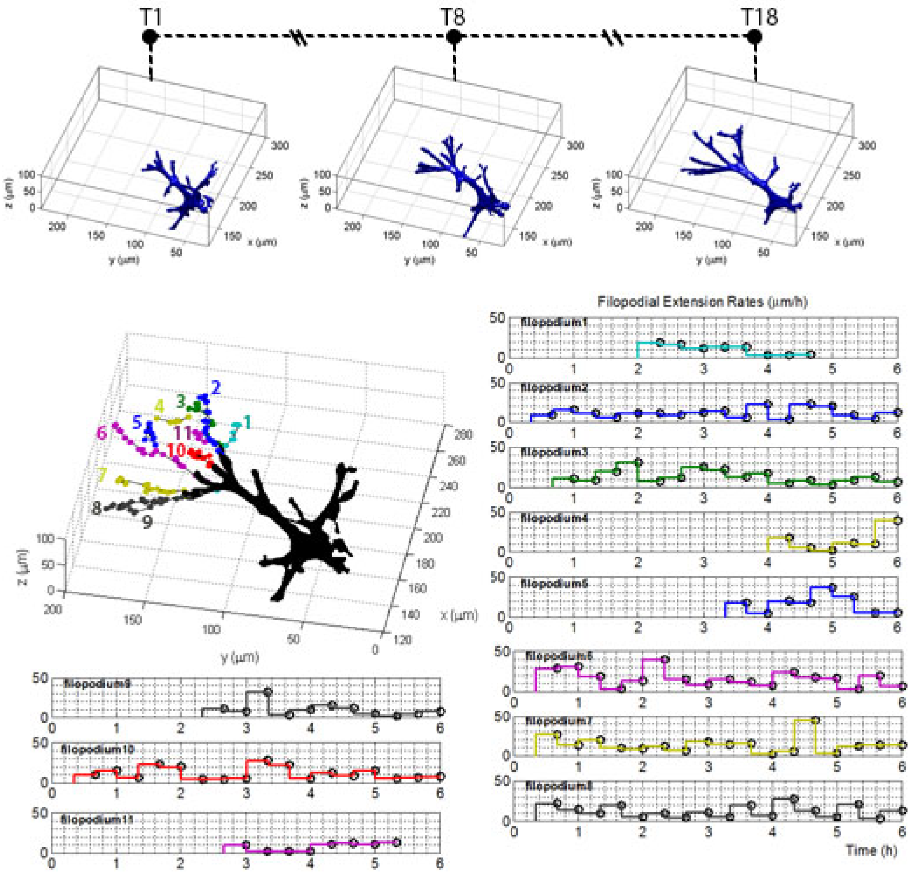

Figure S2. Sprout growth and filopodial extension over the 6-hour period involving the time points T1-T18. The filopoida traced were markd by the numbers alongside them as well as the colors. The filopodial extension rate (unit: μm/h) was considered to be maintained at the same level in each time interval. The time interval T = 20 min.

Collagen Fiber Remodeling

Detection of Collagen Fibers in Microscopy Images. An algorithm was developed to analyze the distribution of collagen fibers and their orientation, which complemented the analysis on the basis of bead displacement. The algorithm was applied to binary images created from high-resolution reflectance images. Figure S3 shows the results of a search for collagen fibers in a reflectance image acquired using the confocal system with 60× oil immersion objective (NA = 1.42). The figure also shows the binary image with collagen fiber pixels indicated in white and the combined image where the searched collagen fibers in red were superimposed onto the original fibers in the binary image. A sprout invading the region is indicated in gray. Four typical regions are highlighted, of which two were located near the leading edges of the tip cell's lamellipodia, and the other two were close to one of the stalk cells. The search results verified the ability of our algorithm to trace the curved collagen fibers with poor connections among their pixels.

Reallocation of Collagen Fibers. The concentration of collagen fibers in a region was highly dependent on their position relative to the sprout interacting with the whole area. A higher concentration usually appeared to surround the head of the sprout and declined with increasing distance from the sprout in a radial direction, as shown in the two pairs of images in Figure S4. Our observation was consistent with the findings reported in [1] about increased collagen density appearing around the periphery of lumens and its further enhancements over time, most likely because of the continued pull actions of the ECs, as suggested by our results from the DVC analysis (Figure 2). These collection actions of the ECs were thought to probably play a role in creating a stiffer structure to support themselves.

Those collagen fibers in the area influenced by the lamellipodia (the estimation of which is discussed in the Results section), in particular for tip cells, may be reallocated depending on lamellipodial activity. During expansion of the lamellipodium, the densifying collagen fibers emerged in its anterior region in preparation for its further expansion, as indicated by the black arrow in Figure S4A. This region marked an increasing density of collagen fibers seen from Figure S4B taken about 13 minutes after capturing Figure S4A. Such an observation again supports the finding that the "pull" behavior is one of the identified interaction behavior types for tip cells. When the lamellipodium retracted, collagen fibers escaped from its region in one direction with an up to 140 μm-long trajectory path, as indicated by the black arrowhead in Figure S4C, compared to that in Figure S4D showing the circumstance about 13 minutes later. This provided evidence of the existence of the identified "release" behavior as another interaction behavior type for tip cells.

Realignment of Collagen Fibers. The sprouts were also capable of aligning the collagen fibers in certain directions, and not just of reallocating them when needed. A clearly visible alignment of the collagen fibers took place near the leading edges of the lamellipodia. They were oriented approximately in line with the longitudinal axis of the neighboring lamellipodium, as indicated in Figure S6A and S6B showing the created rose histogram for regions A and B highlighted in Figure S3. The majority of fibers in region A had been oriented in directions ranging from 35° to 55°, whereas those in region B ranged from 125° to 145°. The longitudinal axis of their neighboring lamellipodium was found to concurrently fall into

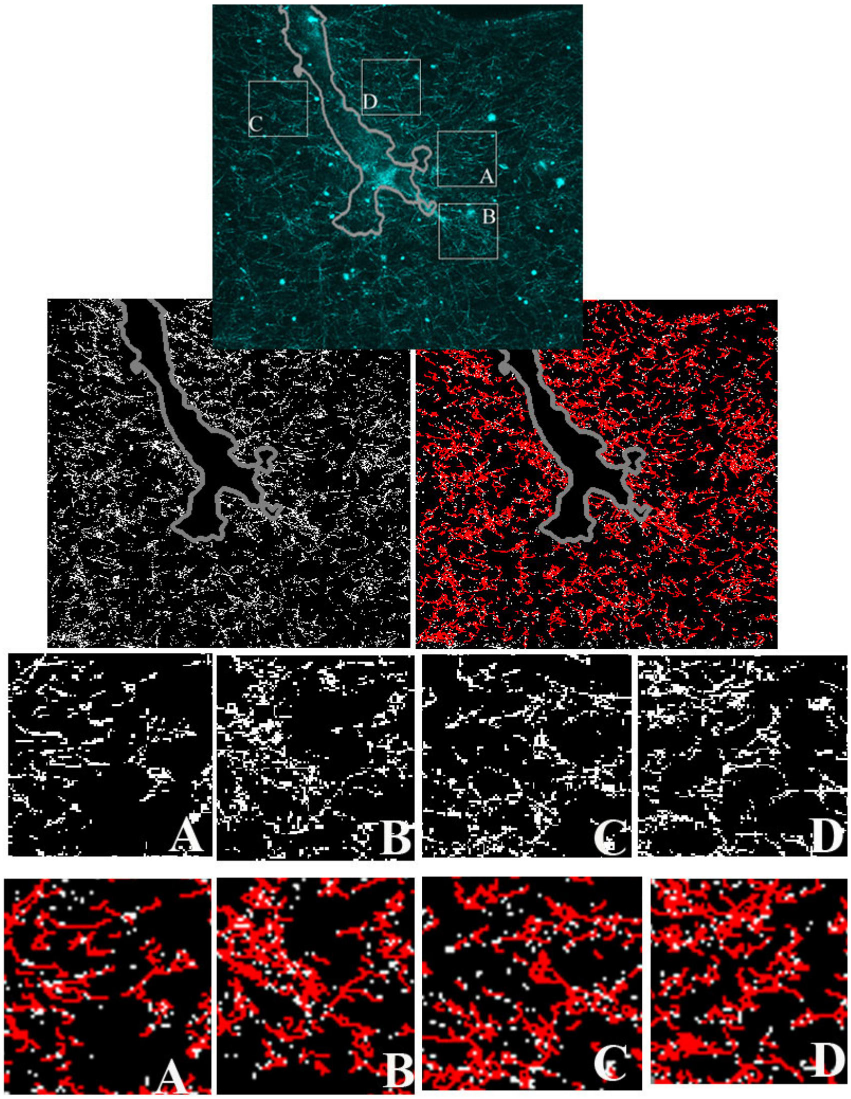

Figure S3. Results of the search for collagen fibers. The top three images show the original reflectance image, its binary image, and the combined image of the searched collagen fibers in red superimposed onto those in the binary image. A sprout that was invading the region is indicated in gray. Four typical regions (A, B, C, and D) are highlighted.

their dominant orientation region. Unlike regions A and B, there was no observable lamellipodium approaching regions C and D; however, the majority of collagen fibers in each of these two regions had been oriented in similar directions, as shown in Figure S6C and S6D. Moreover, their arrangement may correlate with sprout elongation, as manifested by a certain level of consistency between their dominant orientation and the angle of inclination of the sprout.

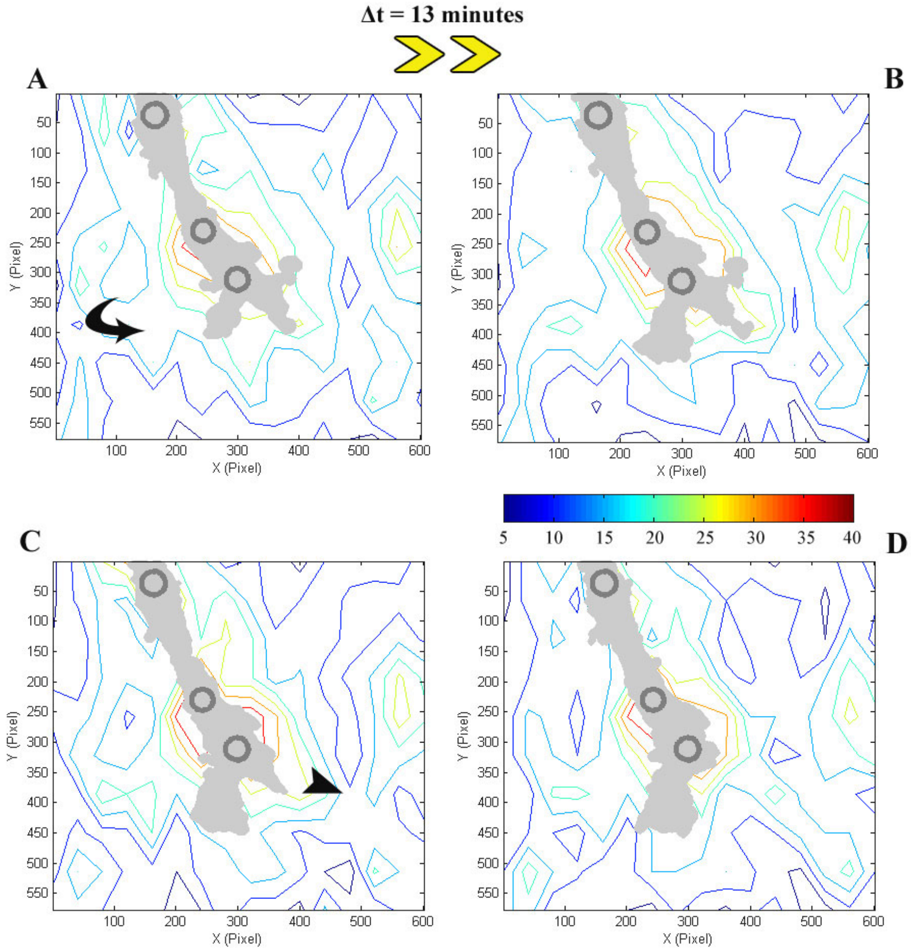

Figure S4. Reallocation of collagen fibers. These figures were generated based on the number of collagen fibers in each rectangular grid. The size of the whole area covered is about 200 μm $\times$200 μm. The outline of a sprout interacting with the area is indicated in grey, and the nuclei of the ECs involved are indicated with circles. (A) and (B) collectively illustrate the densifying of collagen fibers associated with the "pull" behavior of the tip cell's expanding lamellipodium. (C) and (D) show the escape of collagen fibers from the tip cell's retracting lamellipodium in one direction, probably linking to its "release" behavior.

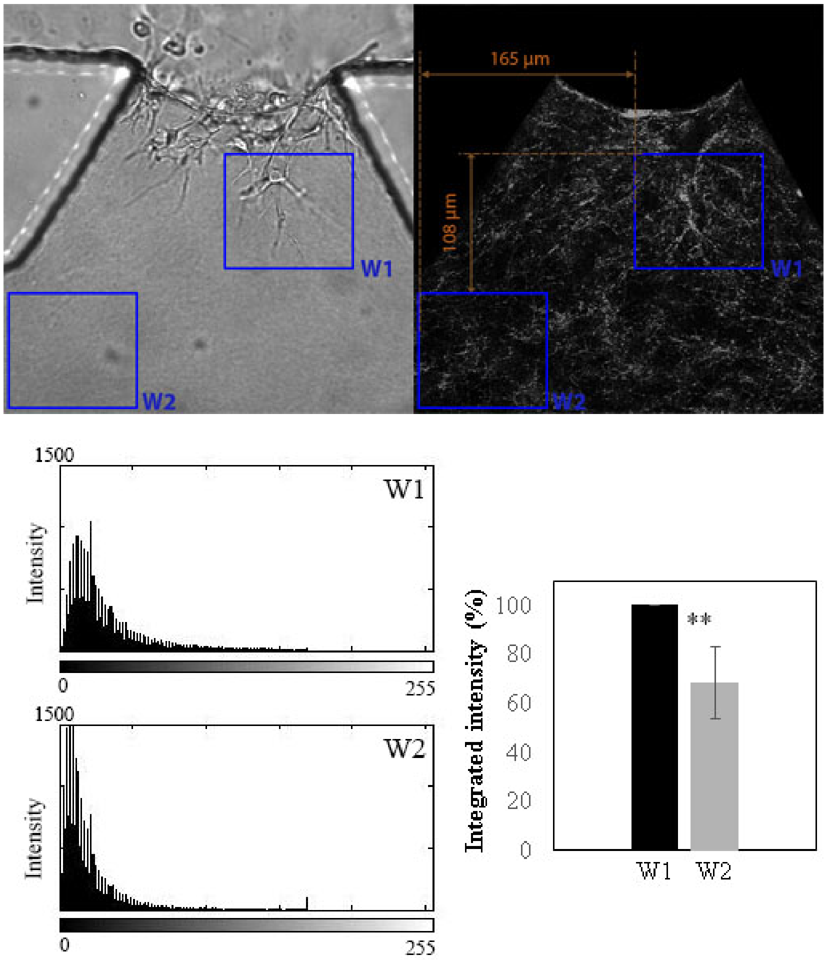

FIGURE S5. Reflectance intensity of collagen fibers in both the occupied and isolated regions. The density of collagen fibers was investigated based on its association with the strength of the reflectance signal. Two regions of 150 pixels $\times$ 150 pixels were selected from the slice (where the center of the target sprout was best represented), one of which was interacting with the sprout. A total of 15 pairs of data points concerning the normalized integrated intensity were obtained for the different sprouts. The result showed that the occupied regions marked a quite significantly higher density of collagen fibers than the unoccupied regions ($P < 0.0001$).

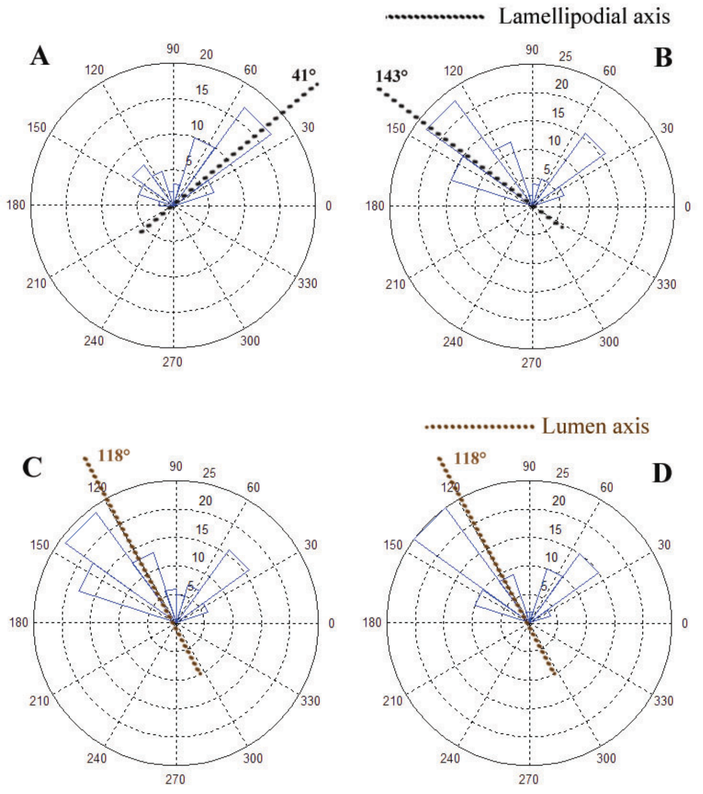

Figure S6. Results of collagen fiber-orientation analysis of four regions. These four regions were selected from the uppermost image shown in Figure S3, of which two (A and B) were located near the leading edges of the tip cell's lamellipodia, and the other two were close to one of the stalk cells. For regions A and B, the longitudinal axis of the neighboring lamellipodium is also marked on the rose histogram, whereas for regions C and D, the lumen axis of both is indicated with a dotted line.

Pattern Recognition of Bead Movement

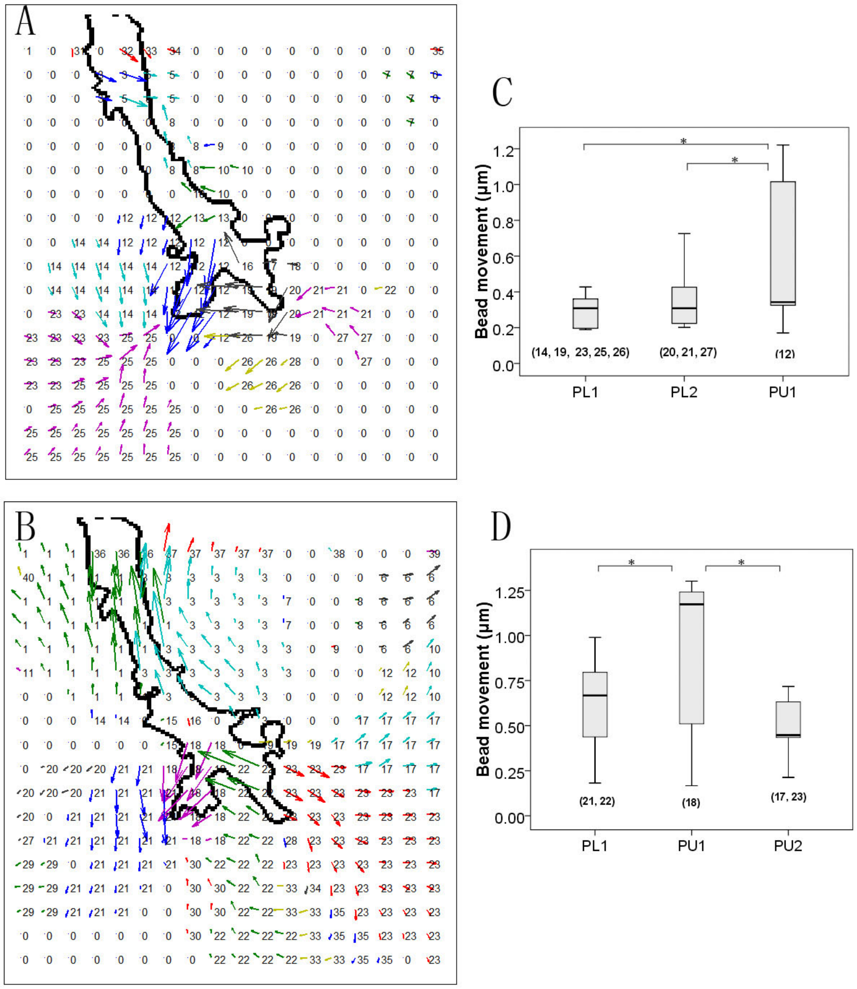

FIGURE S7. Classification of bead movement. Bead movement over a given time period was measured on the chosen slice. The resulting vectors were then classified on the basis of their angle of inclination and positional connectivity. The associated interaction behavior types were determined on the basis of these bead movement groups. In the figure, a group index and a color are allocated to each of the vectors accordingly. In (A), we identified for the tip cell's lamellipodia two pull actions: PL1 (comprising bead groups 14, 19, 23, 25, and 26) and PL2 (20, 21, and 27), and one push action PU1 (12). Comparison of their average strengths is shown in (C). In (B), analysis over a different time period from that in (A) shows one pull action PL1 (21 and 22) and two push actions PU1 (18) and PU2 (17 and 23), the average strengths of which are shown in (D). The differences were significant at $P < 0.05$.

To understand the dynamics of lamellipodial interactions with the ECM, it is necessary to monitor over time the involved interaction behavior types and their

changes in terms of persistence and strength. This was conducted on the basis of the identified interaction behavior types for both tip and stalk cells combined with the features of the associated bead movement.

The central slice used for the targeted lamellipodium was usually selected as that expected to be most representative of the interaction behavior types. Given that two slices were recorded consecutively, the movement of all of the beads included was first established. Scrutiny of the resulting movement vectors was then performed with an algorithm to remove unwanted or meaningless vectors. The remaining vectors were then marked with their angle of inclination, based on which they were further graded into eight angular regions that increased by increments of 45°. The connectivity of vectors falling into the same angular region was examined to ensure those remaining in the same group were also geographically connected. The involved interaction behavior types were finally determined on the basis of these bead movement groups. Illustrations of such characterization can be seen in Figure S7.

References

[1] Lee, P. F., Yeh, A. T. & Bayless, K. J. Nonlinear optical microscopy reveals invading endothelial cells anisotropically alter three-dimensional collagen matrices. *Exp. Cell Res.* **315**, 396-410 (2009).
